# Outcomes of postoperative delirium in patients undergoing cardiac surgery: A systematic review and meta-analysis

**DOI:** 10.3389/fcvm.2022.884144

**Published:** 2022-08-09

**Authors:** Lingyu Lin, Xuecui Zhang, Shurong Xu, Yanchun Peng, Sailan Li, Xizhen Huang, Liangwan Chen, Yanjuan Lin

**Affiliations:** ^1^Department of Nursing, Fujian Medical University, Fuzhou, China; ^2^Department of Cardiac Surgery, Fujian Medical University Union Hospital, Fuzhou, China; ^3^Department of Nursing, Fujian Medical University Union Hospital, Fuzhou, China

**Keywords:** postoperative delirium, outcome, cardiac surgery, mortality, systematic review, meta-analysis

## Abstract

**Background:**

Postoperative delirium (POD) is an acute brain dysfunction that is frequently observed in patients undergoing cardiac surgery. Increasing evidence indicates POD is related to higher mortality among cardiac surgical patients, but the results remain controversial. Moreover, a quantitative evaluation of the influence of POD on hospital days, intensive care unit (ICU) time, and mechanical ventilation (MV) time has not been performed.

**Objective:**

This study aimed to evaluate the correlation between POD and outcomes in patients undergoing cardiac surgery by a systematic review and meta-analysis.

**Materials and methods:**

A total of 7 electronic databases (Cochrane Library, PubMed, EMBASE, CINAHL Complete, MEDLINE, Wan-fang database, and China National Knowledge Infrastructure) were searched from January 1980 to July 20, 2021, with language restrictions to English and Chinese, to estimate the impact of the POD on outcome in patients who underwent cardiac surgery. The meta-analysis was registered with PROSPERO (Registration: CRD42021228767).

**Results:**

Forty-two eligible studies with 19785 patients were identified. 3368 (17.0%) patients were in the delirium group and 16417 (83%) were in the non-delirium group. The meta-analysis showed that compared to patients without POD, patients with POD had 2.77-fold higher mortality (OR = 2.77, 95% CI 1.86–4.11, *P* < 0.001), 5.70-fold higher MV (>24h) rate (OR = 5.70, 95% CI 2.93–11.09, *P* < 0.001); and longer MV time (SMD = 0.83, 95% CI 0.57–1.09, *P* < 0.001), ICU time (SMD = 0.91, 95% CI 0.60–1.22, *P* < 0.001), hospital days (SMD = 0.62, 95% CI 0.48–0.76, *P* < 0.001).

**Conclusion:**

The synthesized evidence suggests that POD is causally related to the increased risk of mortality, prolonged length of ICU and hospital stay, and a longer duration of MV time. Future research should focus on the interventions for POD, to reduce the incidence.

**Systematic review registration:**

[www.crd.york.ac.uk/PROSPERO], identifier [CRD42021228767].

## Introduction

Cardiovascular disease has become one of the greatest threats to human health in the 21st century (1). The number of patients suffering from cardiovascular disease has increased dramatically over recent years worldwide (1, 2), and the amounts of cardiovascular operations have also increased rapidly. According to the latest report, 1.5 million cardiac surgeries are performed globally every year approximately (3), and the incidence of complications varies from 2 to 60% following cardiac surgery (4). POD is the most common complication among cardiac surgical patients with an incidence of 25–52% (5). It is defined as an acute disturbance of consciousness characterized by acute and fluctuating changes in attention, awareness, and cognition (6), with a poor prognosis. An analysis published in Lancet reported that delirium costs more than $164 billion in health care expenses in the United States each year (7), bringing a heavy economic burden to society. Thereby, the prognosis of POD is receiving greater public attention (8).

In recent years, a large body of evidence indicates that POD in patients undergoing cardiac surgery is significantly associated with poor prognosis. However, the results remain controversial. Compared with patients without POD, cardiac surgical patients who develop POD have higher mortality (9, 10). While others suggest that POD is not significantly related to mortality (11, 12). According to our literature search, only one review evaluated the association between POD and mortality in patients undergoing TAVR which was published in 2020 (13), seven studies are included and the sample size is relatively small. Individual studies have insufficient power to detect the association and to persuade convey conflicting results. Furthermore, a quantitative evaluation of the influence of POD on hospital days, ICU time, and MV time has not been performed.

It should be noted that we do not draw enough attention to delirium since the insufficient recognition and under-reporting (14, 15). Knowledge of the true magnitude of POD and its associated burdens in cardiac surgical patients would allow healthcare professionals to allocate much-needed resources toward reducing morbidity and mortality associated with delirium after cardiac surgery. Therefore, we conducted a systematic review and meta-analysis to explore the relationship between POD and outcomes in these patients, including short-term and after-discharge mortality, hospitalization, ICU stays, and MV time, to provide scientific data for POD management after cardiac surgery.

## Materials and methods

### Report and register

This systematic review and meta-analysis followed the PRISMA (16) (see Supplementary Table 1). The protocol has been registered in PROSPERO (Registration: CRD42021228767).

### Data sources and searches

We conducted a comprehensive computerized search of the medical literature using 5 major English databases, including Cochrane Library (search date: August 15, 2021), PubMed (search date: August 25, 2021), EMBASE (search date: August 30, 2021), CINAHL Complete (search date: September 5, 2021), and MEDLINE (search date: September 15, 2021). We also searched two major Chinese databases, Wan-fang database (search date: September 20, 2021), and China National Knowledge Infrastructure (search date: September 20, 2021). All these databases were recommended by domain experts in evidence-based medicine. The retrieval scheme was mainly based on a combination of subject words and free words. Search terms included delirium (i.e., delirium, delirious, intensive care delirium, cognitive dysfunction) and cardiac surgery (i.e., cardiac surgery, heart surgery, open heart; see Supplementary Table 2 for the search strategies). A manual search was further performed to search the reference lists of relevant articles. Databases were searched from January 1980 to July 20, 2021, and the language of studies were limited to English and Chinese.

Studies were included if they satisfied the following inclusion criteria: (1) Type of participants: adult patients (aged ≥18 years) undergo cardiac surgery. (2) Type of exposure: POD, and it must be identified using a validated CAM-ICU, CAM, ICDSC; or diagnosed according to DSM-4 or DSM-5. (3) Type of outcome: studies report at least one of the following outcomes, mortality, hospital days, ICU time, and MV time. If mortality was reported at multiple time points, the longest follow-up mortality was used for analysis. (4) Types of studies: prospective or retrospective observational study. When multiple articles included the same population of patients, only the newest, or the most complete publication was selected. The exclusion criteria were as follows: (1) Conference abstracts and articles where the full text was unavailable. (2) Studies of poor quality (the NOS <5). (3) Repeated published literature.

### Study selection and data extraction

Literature screening was independently conducted by two researchers. First, we used the reference management software Endnote X8 for literature classification, preparation, and removal of duplicates. Then two reviewers independently read titles and abstracts and preliminary screened the literature according to inclusion and exclusion criteria. Finally, the remaining records were evaluated by reading the full-text papers. Reasons for exclusion of studies following full-text reading were recorded. Discrepancies were resolved by discussion or consulting the third reviewer.

Two researchers independently performed the data extraction using a pre-established data extraction table. We recorded the following information (when available): author, publication year, country, study design, sample size, inclusion/exclusion criteria, data collection time, age, gender, types of cardiac surgery, method of POD assessment, clinical outcomes, etc. Authors of studies with missing data were contacted by email to obtain additional data.

### Evaluation of study quality

Study quality was assessed by two researchers using the NOS (17), which included three aspects: object selection, comparability, and exposure/outcome assessment. NOS scores ranged from 0 to 9, and a score of 0 to 4, 5 to 6, and 7 to 9 indicated low, intermediate, and high quality, respectively, (18). The result of the assessment was cross-checked by two researchers and disagreements were resolved under discussion.

Besides, an assessment of the overall quality of evidence was made according to the Grading of Recommendations, Assessment, Development and Evaluation (GRADE) framework (16). We assessed the risk of bias, consistency of effect, indirectness, imprecision, and publication bias. And we used GRADEpro GDT to generate the evidence profile.

### Data synthesis and analysis

All of data were analyzed by the software RevMan5.3., and *P* < 0.05 was considered statistically significant. The outcomes were mortality, hospital days, ICU time, and MV time. When MV time was reported as counting data, we extracted the incidence of MV time (>24h). The inverse variance method with a SMD as the measure of an effect estimate was used for continuous variables, whereas the Mantel–Haenszel method with OR and 95% CI was employed for dichotomous variables.

Before the combined data were analyzed by meta-analysis, the heterogeneity of each group was tested. Heterogeneity was qualified by *I*^2^ (<25%, low heterogeneity; 25–50%, moderate heterogeneity; and >50%, strong heterogeneity). A fixed-effect model was used when the heterogeneity was low or moderate (*P* > 0.1, *I*^2^ < 50%), and a random-effects model was adopted when heterogeneity was high (*P* ≤ 0.1, *I*^2^ ≥ 50%). To explore the source of heterogeneity, we performed subgroup analyses according to the study designs, sample size, countries, types of cardiac surgery et al. And sensitivity analyses were performed by sequentially removing each study and rerunning the analysis, to verify the robustness of the review conclusions. Furthermore, publication bias was measured using a funnel plot.

## Results

### Study selection

**FIGURE 1:**
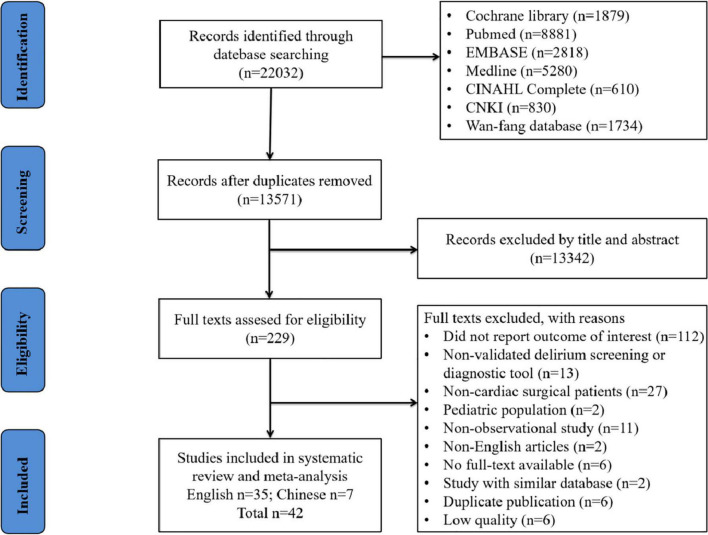
Flow diagram to identify studies reporting the outcome of postoperative delirium in patients undergoing cardiac surgery.

A total of 22,032 records were retrieved from the literature search, and 13571 were obtained after the removal of duplicates. By reading titles and abstracts, 13342 studies were excluded, as they did not fulfill the selection criteria. Eventually, 229 articles were included for full-text review, of which 42 (35 and 7 articles in English and Chinese, respectively) were finally included. The literature screening process was listed in Figure 1.

### Study characteristics

The characteristics of included studies were shown in Table 1. Among the 42 studies, 31 were prospective studies (9, 12, 19–47), and 11 were retrospective studies (10, 48–57), which were published between 2004 and 2021. Sample sizes ranged from 66 to 3397. A total of 19785 patients were included and the male proportion of each study varied from 36.9 to 84.3%. Concerning procedure types, it includes acute CABG (22, 38, 42, 48, 53), valve surgery (10, 21, 26, 31–33, 46, 49, 51), AAD surgery (44, 52, 54, 57), and mixed cardiac surgery (9, 12, 19, 20, 23–25, 27–29, 35, 36, 47, 55, 56).

**TABLE 1:** Characteristics of the included studies.

First author	Country	Study design	Surgery type	Surgery urgency	Sample size	CPB	Age, year	Male, *n* (%)	Surgical risk score	Outcome measurement
Kati Järvelä (9)	Finland	Prospective	Cardiac surgery	Mixed	1036	Mixed	65.7 ± 11.0	765 (73.8)	6.2 ± 3.1 Euroscore	Hospital mortality MV time ICU time
Andrea Kirfel et al. (19)	Germany	Prospective	Cardiac surgery	Elective	254	N/A	70.5 ± 6.4	182 (71.7)	N/A	ICU time Hospital days
Sandra Koster (20)	The Netherlands	Prospective	Cardiac surgery	Elective	300	N/A	70.5 ± 9.3	204 (68.0)	N/A	6-month mortality
Katarzyna Kotfis (12)	Poland	Prospective	Cardiac surgery	Mixed	1797	Yes	72.3 ± 5.7	1161 (64.6)	10.3 ± 11.0 Euroscore logistic	30-day mortality Hospital days ICU time MV time
Kacper Lechowicz (48)	Poland	Retrospective	CABG	Elective	1098	Yes	65.5 ± 9.8	771 (70.2)	4.5 ± 1.0 Euroscore II	1-year mortality Hospital days MV time
Tania Luque (49)	Spain	Retrospective	TAVR	Mixed	501	Yes	82.9 ± 5.8	212 (42.3)	5.9 ± 5.9 Euroscore II	2-year mortality Hospital days
Victor Mauri (21)	Germany	Prospective	TAVR	N/A	661	Yes	82.3 ± 6.6	322 (48.7)	4.0 ± 3.6 Euroscore II	Hospital mortality
Dongliang Mu (22)	China	Prospective	CABG	Elective	243	Yes	61.0 ± 8.3	200 (82.3)	2.6 ± 2.1 Euroscore	MV time
Quyen Nguyen (23)	Canada	Prospective	CABG or valve replacement	Mixed	197	Yes	69.9 ± 11.5	137 (69.5)	1.6 ± 1.6 Euroscore II	Hospital days ICU time
Ieva Norkienë (24)	Lithuania	Prospective	Cardiac surgery	Elective	89	Yes	65.1 ± 10.9	N/A	2.0 ± 1.4 Euroscore II	ICU time MV time
Masato Ogawa (47)	Japan	Prospective	Cardiac surgery	Elective	326	Yes	68.6 ± 14.8	N/A	6.3 ± 2.8 Euroscore II	ICU time
Kamran Shadvar (25)	Iran	Prospective	Cardiac surgery	N/A	200	Mixed	53.3 ± 11.4	N/A	N/A	ICU time MV time
Yukiharu Sugimura (50)	Germany	Retrospective	Cardiac surgery	Mixed	1206	Yes	69.5 ± 11.0	816 (67.7)	N/A	30-day mortality Hospital days
Van der (26)	The Netherlands	Prospective	TAVI	N/A	703	Yes	80.0 ± 6.7	338 (48.1)	13.8 ± 9.4 Logistic Euroscore	3-year mortality Hospital days
Charles H. Brown (27)	America	Prospective	CABG or valve surgery	Elective	66	Yes	69.6 ± 7.4	51 (77.3)	5.6 ± 3.1 Euroscore	Hospital mortality Hospital days ICU time
Hersh S. Maniar (10)	America	Retrospective	TAVR or SAVR	N/A	427	Mixed	74.9 ± 11.1	227 (53.2)	N/A	1-year mortality
Sauër AC (28)	The Netherlands	Prospective	Cardiac surgery	Elective	184	Yes	67.1 ± 11.5	127 (69.0)	4.5 ± 3.8 Euroscore	1-year mortality MV time
Abla Habeeb Allah (29)	Jordan	Prospective	Cardiac surgery	Elective	245	Mixed	58.1 ± 10.6	198 (80.8)	N/A	Hospital days ICU time
Stavros Theologou (30)	Greece	Prospective	Cardiac surgery	Mixed	179	Yes	63.3 ± 12.7	129 (72.1)	4.0 ± 6.0 Euroscore II	Hospital days ICU time MV time
Chetan P. Huded (51)	America	Retrospective	TAVR	N/A	294	N/A	83.0 ± 7.7	151 (51.4)	N/A	30-day mortality Hospital days
Cai et al. (52)	China	Retrospective	AAD surgery	Mixed	301	Yes	50.7 ± 12.2	235 (78.1)	5.6 ± 2.7 Euroscore	Hospital mortality Hospital days ICU time
Sara J Beishuizen (31)	The Netherlands	Prospective	TAVI	N/A	91	Yes	80.9 ± 5.9	37 (40.7)	15.6 ± 6.9 EuroScore logistic	1-year mortality Hospital days
Maciej Bagienski (32)	Poland	Prospective	TAVI	N/A	141	Yes	82.0 ± 1.9	52 (36.9)	14.0 ± 0.1 Euroscore logistic	1-year mortality
Masieh Abawi (33)	The Netherlands	Prospective	TAVR	N/A	268	N/A	80.0 ± 7.0	123 (45.9)	18.0 ± 9.0 Logistic Euroscore	Hospital mortality
Graciela Veliz-Reissmüller (34)	Sweden	Prospective	Cardiac surgery	Elective	107	Yes	71.6 ± 6.0	66 (61.7)	N/A	30-day mortality Hospital days ICU time MV time
Nina Smulter (35)	Sweden	Prospective	Cardiac surgery	N/A	142	Yes	76.6 ± 4.4	92 (64.8)	N/A	ICU time MV time
Silvio Simeone (36)	Italy	Prospective	Cardiac surgery	N/A	89	Yes	89.0 ± 6.9	75 (84.3)	N/A	ICU time MV time
Gianfranco Sanson (37)	Italy	Prospective	Cardiac surgery	Mixed	199	Yes	67.9 ± 10.3	150 (75.4)	N/A	Hospital days ICU time
Franklin Santana Santos (38)	Brazil	Prospective	CABG	Elective	220	Yes	70.7 ± 5.7	142 (64.5)	N/A	Hospital days
Ieva Norkiene (53)	Lithuania	Retrospective	CABG	Mixed	1367	Yes	65.0 ± 9.2	1035 (75.7)	3.6 ± 2.4 Euroscore	Hospital mortality ICU time MV time
Ashok K Kumar (39)	India	Prospective	Cardiac surgery	Mixed	120	Yes	≤60:81 (67.5), >60:39 (32.5)	77 (64.2)	N/A	MV (>24 h)
Jakub Kazmierski (40)	Poland	Prospective	Cardiac surgery	Elective	563	Yes	≥65:247 (43.9)	395(70)	N/A	MV (>24 h)
Yohei Kawatani (54)	Japan	Retrospective	Endovas-cular aortic repair	Elective	81	N/A	74.4 ± 7.9	67 (82.7)	N/A	Hospital days ICU time
Robbert C. Bakker (41)	The Netherlands	Prospective	Cardiac surgery	Elective	201	Yes	76.2 ± 3.8	121 (60.2)	5.6 ± 4.7 Logistic Euroscore	30-day mortality MV (>24 h)
Imran Khan (42)	Pakistan	Prospective	CABG	Elective	735	Yes	55.6 ± 9.7	520 (70.7)	N/A	ICU time MV time
Chaohong Chen (43)	China	Prospective	Cardiac surgery	N/A	276	Mixed	70.6 ± 3.9	192 (69.6)	N/A	ICU time MV time
L H et al. (55)	China	Retrospective	Cardiac surgery	N/A	3397	Yes	60.5 ± 11.5	1939 (57.1)	N/A	MV time
Xianrong Song (44)	China	Prospective	AAD surgery	Mixed	148	Yes	47.7 ± 13.1	99 (66.9)	N/A	Hospital mortality Hospital days ICU time MV time
J W et al. (46)	China	Prospective	Valve replacement	Elective	109	Yes	68.4 ± 5.5	50 (45.9)	N/A	MV time
Qinying Wang (57)	China	Retrospective	Cardiac surgery	N/A	754	Yes	55.2 ± 11.1	485 (64.3)	N/A	Hospital mortality ICU time MV time
Yq et al. (56)	China	Retrospective	AAD surgery	Emergent	152	Yes	50.8 ± 12.8	118 (77.6)	N/A	Hospital days ICU time MV time
Lijing Su (45)	China	Prospective	Cardiac surgery	Mixed	318	Yes	<65:273 (85.8), ≥65:45 (14.2)	186 (58.5)	N/A	MV (> 24h)

Data are presented as n (%) or mean ± standard deviation. CPB, cardiopulmonary bypass; N/A, not applicable; MV, mechanical ventilation; ICU, intensive care unit; CABG, coronary artery bypass graft; TAVR, transcatheter aortic valve replacement; SAVR, surgical aortic valve replacement; TAVI, transcatheter aortic valve implantation; AAD, acute aortic dissection; APACHE, Acute Physiology and Chronic Health Evaluation.

Table 2 displays the screening and morbidity of POD reported by the included studies. The overall incidence of POD was 17.0% (3368 of 19785 patients). As for the assessment tool for POD, a total of 33 studies were reported using a single measurement. Among them, 15 studies used CAM-ICU (10, 21, 22, 25, 30, 36, 39, 41, 44–46, 49, 50, 52, 55), six studies used DMS-4 (26, 31, 38, 40, 42, 53), 2 studies used DMS-5 (12, 57), five studies used ICDSC (9, 24, 37, 47, 54), two studies used CAM (34, 56), two studies used DOS (20, 33), and one study used CHART-DEL (29). Two or more tools were used to diagnose delirium in other studies (19, 23, 27–29, 35, 43, 48, 51).

**TABLE 2:** Postoperative delirium screening and prevalence data from the included studies.

First author	Sample size	No. of patients with POD, *n* (%)	No. of patients without POD, *n* (%)	Pre-existing cognitive or psychological function assessed (assessment method)	Delirium assessment tool	Delirium assessment frequency
Kati Järvelä (9)	1036	119 (11.5)	917 (88.5)	Yes	ICDSC	Daily
Andrea Kirfel et al. (19)	254	127 (50.0)	127 (50.0)	N/A	CAM CAM-ICU 4AT DOS	Every morning
Sandra Koster (20)	300	52 (17.3)	248 (82.7)	N/A	DOS	Three times a day
Katarzyna Kotfis (12)	1797	384 (21.4)	1413 (78.6)	Yes	DSM-5	N/A
Kacper Lechowicz (48)	1098	164 (14.9)	934 (85.1)	Yes	DSM-4 CAM-ICU	Twice a day
Tania Luque (49)	501	110 (22.0)	391 (78.0)	Yes	CAM-ICU	Every 8 hours
Victor Mauri (21)	661	66 (10.0)	595 (90.0)	N/A	CAM-ICU	N/A
Dongliang Mu (22)	243	123 (50.6)	120 (49.4)	Yes	CAM-ICU	Twice daily
Quyen Nguyen (23)	197	44 (22.3)	153 (77.7)	MoCA	CAM CAM-ICU	Every 4 hours in the ICU/every 8 hours on the hospital wards
Ieva Norkienë (24)	87	12 (13.3)	75 (86.2)	MMSE	ICDSC	Every 8 hours
Masato Ogawa (47)	326	43 (13.2)	283 (86.8)	N/A	ICDSC	Every 8 hours
Kamran Shadvar (25)	200	47 (23.5)	153 (76.5)	N/A	CAM-ICU	N/A
Yukiharu Sugimura (50)	1206	140 (11.6)	1066 (88.4)	N/A	CAM-ICU	Every 8 hours
Van der (26)	703	116 (16.5)	587 (83.5)	Yes	DSM-4	Three times a day
Charles H. Brown (27)	66	37 (56.1)	29 (44.0)	MMSE	CAM CAM-ICU	N/A
Hersh S. Maniar (10)	427	135 (31.6)	292 (68.4)	Yes	CAM-ICU	Twice daily
Sauër AC (28)	184	23 (12.5)	161 (87.5)	Yes	CAM CAM-ICU	Twice daily
Abla Habeeb Allah (29)	245	22 (9.0)	223 (91.0)	Yes	brief CAM CAM-ICU	Daily
Stavros Theologou (30)	179	20 (11.2)	159 (88.8)	N/A	CAM-ICU	Twice every nursing shift
Chetan P. Huded (51)	294	61 (20.7)	233 (79.3)	Yes	CAM-ICU CAM	Twice daily
Cai et al. (52)	301	73 (24.3)	228 (75.7)	N/A	CAM-ICU	N/A
Sara J Beishuizen (31)	91	14 (15.4)	77 (84.6)	MMSE	DSM-4	N/A
Maciej Bagienski (32)	141	29 (20.6)	112 (79.4)	Yes	CHART-DEL	N/A
Masieh Abawi (33)	268	36 (13.4)	232 (86.6)	Yes	DOS	N/A
Graciela Veliz-Reissmüller (34)	107	25 (23.4)	82 (76.6)	MMSE	CAM	Daily
Nina Smulter (35)	142	78 (54.9)	64 (45.1)	MMSE	MMSE OBS	N/A
Silvio Simeone (36)	89	65 (73.0)	24 (27.0)	N/A	CAM-ICU	Daily
Gianfranco Sanson (37)	199	61 (30.7)	138 (69.3)	N/A	ICDSC	Three times a day
Franklin Santana Santos (38)	220	74 (33.6)	146 (66.4)	MMSE GDS	DSM-4	Daily
Ieva Norkiene (53)	1367	42 (3.1)	1325 (96.9)	Yes	DSM-4	N/A
Ashok K Kumar (39)	120	21 (17.5)	99 (82.5)	CAM	CAM-ICU	Daily
Jakub Kazmierski (40)	563	92 (16.3)	471 (83.7)	MMSE	DSM-4	Daily
Yohei Kawatani (54)	81	20 (24.7)	61 (75.3)	N/A	ICDSC	N/A
Robbert C. Bakker (41)	201	63 (31.3)	138 (68.7)	MMSE HADS	CAM-ICU	Daily
Imran Khan (42)	735	161 (21.9)	574 (78.1)	MMSE	DSM-4	N/A
Chaohong Chen (43)	276	98 (35.5)	178 (64.5)	N/A	CAM CAM-ICU	Twice daily
L H et al. (55)	3397	186 (5.5)	3211 (94.5)	N/A	CAM-ICU	Twice daily
Xianrong Song (44)	148	46 (31.1)	102 (68.9)	Yes	CAM-ICU	Three times a day
J W (46)	109	33 (30.3)	76 (69.7)	Yes	CAM-ICU	Twice daily
Qinying Wang (57)	754	158 (21.0)	596 (79.0)	Yes	DSM-5	N/A
Qianyue Zhu, (56)	152	55 (36.2)	97 (63.8)	N/A	CAM	Daily
Lijing Su (45)	318	93 (29.2)	225 (70.8)	N/A	CAM-ICU	Twice daily

POD, postoperative delirium; ICDSC, Intensive Care Delirium Screening Checklist; CAM, Confusion Assessment Method; CAM-ICU, Confusion Assessment Method for ICU; 4 AT, 4 ‘A’s Test; DOS, Delirium Observation Scale; DSM, Diagnostic and Statistical Manual of Mental Disorders; N/A, not applicable; ICU, intensive care unit; MMSE, Mini-mental State Examination; OBS, Organic Brain Syndrome Scale; GDS, Geriatric Depression Scale; HADS, Hospital Anxiety and Depression Scale.

### Assessment of study quality

The quality of included studies was assessed using the NOS quality scale. Three domains were assessed: selection, comparability, and outcome. The results of the quality assessment are shown in Supplementary Table 3. All included studies scored greater than four points, 31 studies were classified as high-quality, and 11 studies were classified as moderate-quality.

### Association of POD with clinical outcomes

#### Mortality

**FIGURE 2:**
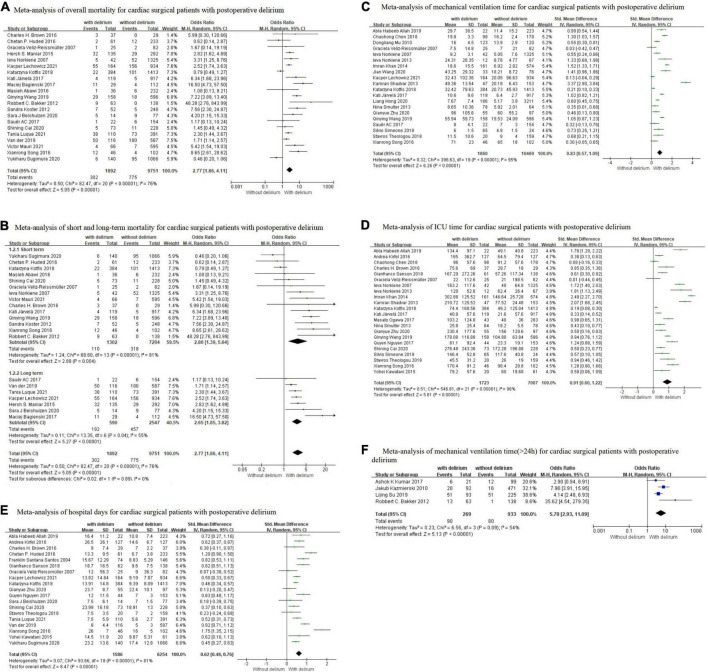
Results of meta-analysis on the association between postoperative delirium and outcomes **(A)** Overall mortality; **(B)** Short-term and long-term mortality; **(C)** Mechanical ventilation time; **(D)** ICU time; **(E)** Hospital days; **(F)** Prolonged mechanical ventilation time (>24h). The summary effects were obtained using a random-effects model. The size of the data markers indicates the weight of the study. The diamond data markers indicate pooled ORs or SMD, and 95% CI.

Twenty-one studies (9, 10, 12, 20, 21, 26–28, 31–34, 41, 44, 48–53, 57) reported the incidence of mortality, which included 11643 individuals. The results of a random-effects model showed that patients with POD had 2.77-fold mortality compared to those without POD (OR = 2.77, 95% CI 1.86–4.11, *P* < 0.001), with a significant heterogeneity (*I^2^* = 76%; Figure 2A). Furthermore, we analyzed mortality based on different time points (short term ≤6 months and long term >6 months). The pooled results showed that there was a significant association between POD and short-term mortality (OR = 2.80, 95% CI 1.39–5.64, *P* = 0.004; *I*^2^ = 81%) and long-term mortality (OR = 2.65, 95% CI 1.86–4.11, *P* < 0.001; *I*^2^ = 76%), with a low heterogeneity between two groups (*I^2^* = 0%; Figure 2B).

To explore the sources of heterogeneity, subgroup analyses for mortality were conducted by the study design, sample size, male proportion, surgery type, and study region as shown in Table 3. When subgroups were stratified by study design, we found a significant effect between prospective studies (OR = 3.81, *P* < 0.001) and retrospective studies (OR = 2.12, *P* = 0.004). When subgroups were stratified by sample size, the mortality was significantly higher in patients with POD in the <500 subgroups (OR = 3.60, *P* < 0.001) and ≥500 subgroups (OR = 2.26, *P* = 0.002). When subgroups were stratified by male proportion, the risk of mortality was higher in the <50% subgroup (OR = 3.20, *P* < 0.001) than in the 50–70% subgroup (OR = 2.47, *P* = 0.030). When subgroups were stratified by surgery type, summary effects were only statistically significant in mixed cardiac surgery (OR = 2.93, *P* = 0.040), valve surgery (OR = 2.72, *P* < 0.001), and CABG (OR = 2.60, *P* < 0.001), whereas no significant was found in aortic surgery subgroup. When subgroups were stratified by region, the mortality was significantly higher in the Asia subgroup (OR = 4.63, *P* = 0.004) and Europe subgroup (OR = 2.63, *P* < 0.001), but not in the America subgroup. The forest plots are presented in Supplementary Figure 1. It was discovered that the results of each subgroup analysis were consistent with the overall results, but the between-study heterogeneity within subgroups remained substantial. After excluding five studies (12, 26, 32, 50, 57), the heterogeneity decreased substantially and the result of each subgroup was not significantly changed.

**TABLE 3:** Subgroup analysis of pooled OR for mortality.

Categories	No. of studies	No. of patients	Pooled OR (95% CI)		Heterogeneity	
			Random	*P*-value	*I*^2^ (%)	*P*-value
Study design	21	9751	2.67 (1.84, 3.89)	<0.001	75	<0.001
Prospective	13	4686	3.48 (1.93, 6.29)	<0.001	75	<0.001
Retrospective	8	5065	2.12 (1.27, 3.53)	0.004	78	<0.001
Sample size	21	9751	2.67 (1.84, 3.89)	<0.001	75	<0.001
< 500	12	1927	3.6 (1.97, 6.59)	<0.001	54	0.010
≥ 500	9	7824	2.16 (1.34, 3.49)	0.002	84	<0.001
Male proportion	21	9751	2.47 (1.84, 3.89)	<0.001	75	<0.001
< 50%	6	1994	2.81 (1.71, 4.60)	<0.001	63	0.020
50%-70%	10	4324	2.47 (1.10, 5.58)	0.030	86	<0.001
> 70%	5	3433	2.63 (1.92, 3.61)	<0.001	0	0.490
Surgery type	21	9751	2.67 (1.84, 3.89)	<0.001	75	<0.001
Cardiac surgery	9	4643	2.93 (1.07, 8.01)	0.040	86	<0.001
Aortic surgery	2	330	3.48 (0.60, 20.04)	0.160	79	0.030
CABG	2	2259	2.60 (1.85, 3.67)	<0.001	0	0.610
Valve surgery	8	2519	2.55 (1.69, 3.85)	<0.001	58	0.020
Region	21	9751	2.67 (1.84, 3.89)	<0.001	75	<0.001
Europe	16	8300	2.50 (1.64, 3.82)	<0.001	74	<0.001
Asia	3	926	4.63 (1.65, 13.02)	0.004	72	0.030
America	2	525	1.58 (0.37, 6.68)	0.540	70	0.070

CABG, coronary artery bypass graft; OR, odds ratio; CI, confidence interval.

#### Mechanical ventilation time

Twenty studies (9, 12, 22, 24, 25, 28–30, 34–36, 42, 47, 48, 53) reported the MV time as an outcome measure, which included 13503 individuals. Using a random-effects model, the pooled SMD was 0.83 (SMD = 0.83, 95% CI 0.57–1.09, *P* < 0.001) with significant heterogeneity (*I^2^* = 95%), which showed that patients with POD had significantly longer MV time compared to those without POD (Figure 2C). Findings from subgroup analysis showed that the MV time was longer in patients aged <60 (SMD = 1.27, 95% CI 0.65–1.89, *P* < 0.001), and the studies with male proportion <60% (SMD = 0.98, 95% CI 0.18–1.79, *P* = 0.020). Nevertheless, the subgroup analysis still showed considerable heterogeneity (> 90%; Supplementary Table 3). Sensitivity analysis also failed to find the source of heterogeneity.

#### Intensive care unit time

Twenty-two studies (9, 12, 19, 23–25, 27, 29, 30, 34, 47, 52, 53, 56) reported the ICU time as an outcome measure, which included 9231 individuals. The results with a random-effects model showed that the ICU time was significantly longer for patients with POD than for those without POD patients (SMD = 0.91, 95% CI 0.60–1.22, *P* < 0.001) with significant heterogeneity (*I*^2^ = 96%; Figure 2D). The subgroup analysis showed that the ICU time was longer in elective surgery patients (SMD = 1.10, 95% CI 0.38–1.83, *P* = 0.003), and the Asia population (SMD = 1.13, 95% CI 0.46–1.62, *P* < 0.001). Study design, sample size, operation time, and study region were not sources of heterogeneity because heterogeneity was still high after subgroup analysis (Supplementary Table 4). Furthermore, the sensitivity analysis did not find any study that significantly affected heterogeneity.

#### Hospital days

Nineteen studies (12, 19, 23, 26, 27, 29–31, 47–52) reported the length of hospital days as an outcome measure, which included 7840 individuals. The results with a random-effects model showed that the hospital days of the delirium group were 0.62 days longer than those without delirium (SMD = 0.62, 95% CI 0.48–0.76, *P* < 0.001) with significant heterogeneity (*I*^2^ = 81%; Figure 2E). The subgroup analysis showed that the hospital days was longer in the valve surgery patient (SMD = 0.75, 95% CI 0.39–1.11, *P* < 0.001), patient >60 (SMD = 0.75, 95% CI 0.39–1.11, *P* < 0.001), and the studies with male proportion <60 (SMD = 0.75, 95% CI 0.39–1.11, *P* < 0.001). It was discovered that the results of each subgroup analysis were consistent with the overall results, but the between-study heterogeneity within subgroups remained substantial (Supplementary Table 5). After excluding four studies (26, 37, 44, 51), the heterogeneity decreased substantially and the result of each subgroup was not significantly changed.

#### Mechanical ventilation time (>24 h)

Four studies (39–41, 45) reported the incidence of MV (>24h) as an outcome measure, which included 1202 individuals. The pooled OR using a random-effects model was 5.70 (95% CI 2.93–11.09, *P* < 0.001) with moderate heterogeneity (*I^2^* = 54%), which showed that patients with POD had a 5.7-fold incidence of MV (>24h) compared to those without POD (Figure 2F). The sensitivity analysis showed that heterogeneity was evidently reduced (*I*^2^ = 34%, *P* = 0.22) after excluding Bakker et al.’s study (41), which may be due to the difference in age of the study population.

### Publication bias

**FIGURE 3:**
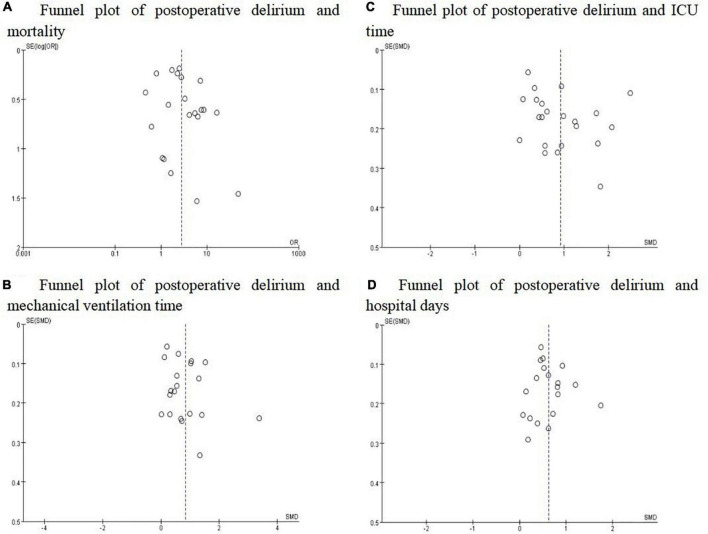
Funnel plots for the studies involved in the meta-analysis. **(A)** Mortality; **(B)** Mechanical ventilation time; **(C)** ICU time; **(D)** Hospital days. The distribution was not completely symmetrical around the funnel plot, which suggested the possibility of publication bias.

To assess potential publication bias, the tendency that significant results are more likely to be published than negative results, we examined each outcome by funnel plot. As shown in Figure 3, a certain degree of asymmetry was observed, which indicated slight publication bias.

#### Grading of recommendations, assessment, development and evaluation of certainty of findings

**FIGURE 4:**
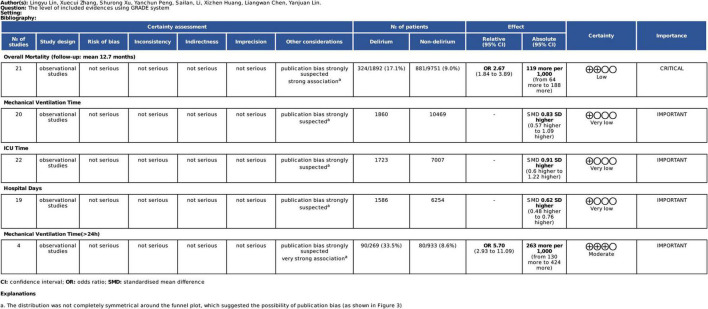
Grading of recommendations, assessment, development and evaluation (GRADE) summary of findings table.

Based on the GRADE approach, the evidence quality of overall mortality was low, and the evidence quality of MV time, ICU time, and hospital days were very low. Besides, we found a moderate quality of evidence for MV time (>24h) (as shown in Figure 4).

## Discussion

The present systematic review and meta-analysis identified 42 studies enrolling a total of 19,785 patients, to summarize the relationship between POD and outcomes in patients undergoing cardiac surgery. The overall incidence of POD was 17.0%, and the results of the meta-analysis showed POD has been associated with increased mortality, longer duration of MV time, ICU stays, and hospitalization among cardiac surgical patients. Given certain heterogeneity among studies, we next conducted subgroup analysis based on study design, sample size, male proportion, surgery type, study region, etc. Despite remaining heterogeneity after subgroup analysis, it was partly reduced in some subgroups. This suggests that POD is a very common and severe neuropsychiatric syndrome, that seriously affects the prognosis of patients.

In this study, POD was significantly linked to mortality. However, due to the significant heterogeneity across studies, this relationship may be limited. Subgroup analyses were conducted based on the timing of mortality, and the results indicated that POD is related to short- and long-term mortality of cardiac surgery patients, which was partially consistent with Crocker et al. (58). The difference was that Crocker et al. indicated that POD was no significant association with short-term mortality. This may be linked to no meta-regression analysis performed to assess the influence of POD on short-term mortality in their study, as only two of the included studies had reported 6-month mortality. However, short-term mortality was reported in 13 studies in our article, where a larger sample size may yield different results.

The mechanism by which POD increases mortality risk is not understood. We propose the following explanations to comprehend the results. First, higher levels of postoperative pro-inflammatory cytokines (e.g., IL-2, IL-6, TNF-α, MCP-1) were associated with a higher risk to develop POD in cardiac surgery patients (59, 60). Elevated levels of pro-inflammatory cytokines reflect an active inflammatory response in the body, which may cause systemic inflammatory response syndrome. Systemic inflammation can alter the brain’s inflammatory status, produce acute cognitive impairments, such as POD, and drive new pathology and accelerated decline (61). Thus, the presence of POD can be considered a marker of hyperinflammatory conditions, which are associated with higher mortality (62, 63). Second, Holmes et al. (64) indicated that POD may represent an extreme non-adaptive presentation of sickness. Patients who develop delirium after cardiac surgery could cause cognitive impairment, decreased consciousness, behavioral abnormalities, etc., which increase cerebrovascular accidents, bleeding, infection, and other complications risk (50, 65, 66), and patients with hypoactive motor-type delirium may present with more severe systemic disease, increased complications of inactivity (e.g., dehydration, pressure ulcers, hypoventilation, and venous thrombosis; 67), these complications are associated with higher mortality (68). Finally, POD is closely associated with the presence of hemodynamic or electrical instability, and disorders of fluids and electrolytes, which may increase the risk of mortality (69). The accidental extubation, difficulty in weaning, or reintubation in patients with MV were also increased (70–73), which required an increased duration of MV, and it has been well documented that prolonged MV time is an independent predictor of increased mortality (74). The data also showed that POD could result in prolonged hospital and ICU stays of the patients, concordant with the results of Salluh’s study (8). The longer the patients stay in the hospital, the more they are at risk of complications and death. All these factors may explain the increased mortality risk among patients who develop POD. Further investigation regarding the pathophysiological mechanism of POD is still warranted to fully understand the reasons why POD led to poor outcomes among cardiac surgical patients.

Given the poor outcomes among cardiac surgery patients who developed POD, there exists a great opportunity to improve the outcomes among these patients. A review (75) in Lancet reported that 30 to 40% of delirium may be prevented by early detection, and takes pharmacologic or non-pharmacologic interventions. Current guidelines (76) recommend using a multicomponent, non-pharmacologic intervention to reduce delirium. The strategies include cognitive training, improving sleep quality, improving wakefulness, early rehabilitation, etc. (conditional recommendation, low quality of evidence). However, it is still uncertain as to which interventions result in the effect. In the future, emphasis should be put on improving the awareness of medical staff on delirium, and undertaking studies to validate the intervention effects, to provide widely applicable evidence for healthcare policymakers.

There was a high degree of heterogeneity observed in our meta-analysis. The reasons might be as follows: first, the methods for diagnosing delirium were different. The incidence of delirium may be dependent on the different diagnostic criteria applied, different tools used, and different evaluators. Second, the study periods of the included studies were different. With the progressive developments in delirium research, the attention toward POD has gradually increased. In previous years, POD has not yet attracted enough attention from medical staff, and there is also wide variation across hospitals in the treatment of POD. In addition, sample sources were different. The structure of the population in different studies was different such as age, gender ratio, race, disease severity, and surgery types, which could contribute to the different clinical outcomes. Most of the included studies did not provide adjusted data due to the high risk of confounding bias, for example, age, sex, and disease severity, which is the reason why we did not use the adjusted data for further analysis. Regarding the mortality outcome, we conducted a sensitivity analysis. The heterogeneity was reduced from 75 to 24% after the removal of five studies (12, 26, 32, 50, 57), which indicates that the five studies were the source of heterogeneity.

The present meta-analysis exhibited several strengths, compared to the previously published meta-analysis (13, 58). In the first instance, we used a robust methodology following PRISMA guidelines and a comprehensive search strategy, to ensure the inclusion of all relevant literature. Second, we included 42 studies with a larger sample size. It could provide high statistical power to quantitatively evaluate the association between POD and clinical outcomes. Hence, the validity of the results is more reliable. In addition, the included studies in this meta-analysis had high NOS scores which were strictly following the inclusion and exclusion criteria, thus, reducing the potential selection bias.

However, there remain limitations in this study as well. First, a meta-analysis of MV time, and ICU time showed heterogeneity, but sensitivity analysis and subgroup analysis failed to eliminate it. The random-effects model is used for data processing, which may have a slight impact on the reliability of the results. We speculate that heterogeneity might be partially explained by the differences in factors such as patient characteristics (age, sex, type of surgery, etc.), different diagnostic criteria for delirium, unequal levels of regional medical care, and frequency of delirium assessment. Second, variations in the assessment tools and the assessment time-points of delirium might affect the results. Delirium is a fluctuation in mental status that can change over time and may have occurred before or after assessments. Thus, the true incidence of POD and its effects on clinical outcomes might be underestimated. All included studies screened patients utilizing validated delirium assessment tools, but the latest research states that POD needs to meet DSM-5 diagnostic criteria (13), and not all studies achieve this. Future prospective studies with standardized delirium assessment methods are still needed to detect delirium accurately and reliably. Third, due to insufficient data, we could not further evaluate other potential factors that may affect the heterogeneity between studies, such as Euroscore score, complications, and the use of anesthetic drugs. Finally, publication bias remains a major concern for all kinds of meta-analyses because non-significant or negative results are less likely to be published than studies with positive and significant results. To comprehensively identify negative or insignificant outcomes, we used delirium and cardiac surgery as keywords which meant the kinds of literature published on this topic were eligible, to ensure we identified as many relevant studies as possible; and also incorporated all reported outcome measures from each study. In addition, funnel plots were constructed to assess potential publication bias, and it is worth mentioning that there was no observable publication bias.

## Conclusion

In this meta-analysis, we found that POD was involved in poor prognosis among cardiac surgical patients. Patients who develop POD exhibit longer MV time, ICU stay, hospital stay, and greater risk of mortality than patients without POD. Future research should focus on developing and testing interventions for delirium, to reduce its incidence and thereby lower the risk of adverse outcomes in these patients.

## Data availability statement

The original contributions presented in this study are included in the article/Supplementary material, further inquiries can be directed to the corresponding author/s.

## Author contributions

LL: project administration, conceptualization, investigation, date curation, methodology, formal analysis, and writing—original draft, review and editing. XZ: conceptualization, investigation, date curation, formal analysis, and writing—original draft, review and editing. SX: methodology, date curation, and writing—original draft, review and editing. YP: conceptualization and writing—original draft, review and editing. SL: software, date curation, and writing—review and editing. XH: date curation and writing—review and editing. LC: supervision and funding acquisition. YL: conceptualization, supervision, and funding acquisition.

## Abbreviations

POD: postoperative delirium
ICU: intensive care unit
MV: mechanical ventilation
PRISMA: Preferred Reporting Items for Systematic Reviews and Meta-Analyses
CAM-ICU: Confusion Assessment Method for ICU
CAM: Confusion Assessment Method
ICDSC: Intensive Care Delirium Screening Checklist
DSM: Diagnostic and Statistical Manual of Mental Disorders
NOS: Newcastle-Ottawa scale
SMD: standardized mean difference
OR: odds ratio
CI: confidence interval
CABG: coronary artery bypass graft
AAD: acute aortic dissection
DOS: Delirium Observation Screening Scale
CHART-DEL: Chart-Based Delirium Identification Instrument
CPB: cardiopulmonary bypass
N/A: not applicable
TAVR: transcatheter aortic valve replacement
SAVR: surgical aortic valve replacement
TAVI: transcatheter aortic valve implantation
APACHE: Acute Physiology and Chronic Health Evaluation
4 ‘AT’: 4 ‘A’s test
MMSE: Mini-Mental State Examination
OBS: Organic Brain Syndrome Scale
GDS: Geriatric Depression Scale
HADS: Hospital Anxiety And Depression Scale.
