# Supplementary material for: Outcomes of postoperative delirium in patients undergoing cardiac surgery: A systematic review and meta-analysis
Source: Front Cardiovasc Med. 2022 Aug 9;9:884144. doi: 10.3389/fcvm.2022.884144 (PMC9395738; doi:10.3389/fcvm.2022.884144)
Supplement: Supplementary file 1 [file Data_Sheet_1.docx]

**Supplementary Table S1 Search strategy for each database**

| **Database** | **Search strategies** | **Search date** | **Search results** |
| --- | --- | --- | --- |
| **Cochrane Library** | #1 MeSH descriptor: [Delirium] explode all trees  #2 (delirious):ti,ab,kw OR (delusion):ti,ab,kw OR (delusions):ti,ab,kw OR (postoperative delirium):ti,ab,kw OR (intensive care delirium):ti,ab,kw OR (intensive care unit syndrome):ti,ab,kw OR (intensive care delirium psychosis):ti,ab,kw OR (ICU syndrome):ti,ab,kw OR (ICU psychosis):ti,ab,kw OR (ICU delirium):ti,ab,kw OR (acute brain syndrome):ti,ab,kw OR (acute confusional syndrome):ti,ab,kw OR (acute confusion):ti,ab,kw OR (confusion state):ti,ab,kw OR (cognitive impairment):ti,ab,kw OR (cognitive dysfunction):ti,ab,kw OR (psychomotor agitation):ti,ab,kw OR (psychotic disorders):ti,ab,kw (Word variations have been searched)  #3 #1 OR #2  #4 MeSH descriptor: [coronary artery bypass] explode all trees  #5 (cardiopulmonary bypass):ti,ab,kw OR (aortocoronary bypass):ti,ab,kw OR (coronary surgery):ti,ab,kw OR (CABG):ti,ab,kw OR (open heart):ti,ab,kw OR (heart operation):ti,ab,kw OR (heart surgery):ti,ab,kw OR (heart surgical procedure):ti,ab,kw OR (cardiac surgery):ti,ab,kw OR (cardiac operation):ti,ab,kw OR (cardiac surgical procedures):ti,ab,kw OR (cardiovascular surgical procedure):ti,ab,kw OR (valv* surgery):ti,ab,kw OR (valve replacement):ti,ab,kw OR (congenital heart):ti,ab,kw OR (aortic dissection):ti,ab,kw OR (aneurysm):ti,ab,kw (Word variations have been searched)  #6 #4 OR #5  #7 #3 AND #6 | **August 15, 2021** | **1879** |
| **PubMed** | #1 (delirium[MeSH Terms]) OR (delirious) OR (delusion) OR (delusions) OR (postoperative delirium) OR (intensive care delirium) OR (intensive care unit syndrome) OR (intensive care delirium psychosis) OR (ICU syndrome) OR (ICU psychosis) OR (ICU delirium) OR (acute brain syndrome) OR (acute confusional syndrome) OR (acute confusion) OR (confusion state) OR (cognitive impairment) OR (cognitive dysfunction) OR (psychomotor agitation) OR (psychotic disorders)  #2 (coronary artery bypass [MeSH Terms]) OR (cardiopulmonary bypass) OR (aortocoronary bypass) OR (coronary surgery) OR (CABG) OR (open heart) OR (heart operation) OR (heart surgery) OR (heart surgical procedure) OR (cardiac surgery) OR (cardiac operation) OR (cardiac surgical procedures) OR (cardiovascular surgical procedure) OR (valv* surgery) OR (valve replacement) OR (congenital heart) OR (aortic dissection) OR (aneurysm)  3 #1 AND #2 | **August 25, 2021** | **8881** |
| **EMBASE** | #1 ('delirium')/exp OR ('delirious'):ab,ti OR ('delusion?'):ab,ti OR ('postoperative delirium'): ab,ti OR ('intensive care delirium':ab,ti) OR ('intensive care unit syndrome':ab,ti) OR ('intensive care delirium psychosis':ab,ti ) OR ('ICU syndrome'):ab,ti OR ('ICU psychosis'):ab,ti OR ('ICU delirium'):ab,ti OR ('acute brain syndrome'):ab,ti OR ('acute confusional syndrome'):ab,ti OR ('acute confusion'):ab,ti OR ('confusion state'):ab,ti OR ('cognitive impairment'):ti,ab OR ('cognitive dysfunction'):ti,ab OR ('psychomotor agitation'): ab,ti OR ('psychotic disorders'): ab,ti  #2 ('coronary artery bypass')/exp OR ('cardiopulmonary bypass'): ab,ti OR ('aortocoronary bypass'): ab,ti OR ('coronary surgery'): ab,ti OR ('CABG'): ab,ti OR ('open heart'): ab,ti OR ('heart operation'): ab,ti OR ('heart surgery'): ab,ti OR ('heart surgical procedure'): ab,ti OR ('cardiac surgery'): ab,ti OR ('cardiac operation'): ab,ti OR ('cardiac surgical procedures'): ab,ti OR ('cardiovascular surgical procedure'): ab,ti OR ('valv* surgery'): ab,ti OR ('valve replacement'): ab,ti OR ('congenital heart'): ab,ti OR ('aortic dissection'): ab,ti OR ('aneurysm'): ab,ti  3 #1 AND #2 | **August 30, 2021** | **2818** |
| **CINAHL Complete** | #1 MH 'Delirium'  #2 (AB delirious) OR (AB delusion) OR (AB delusions) OR (AB postoperative delirium) OR (AB intensive care delirium) OR (AB intensive care unit syndrome) OR (AB intensive care delirium psychosis) OR (AB ICU syndrome) OR (AB ICU psychosis) OR (AB ICU delirium) OR (AB acute brain syndrome) OR (AB acute confusional syndrome) OR (AB acute confusion) OR (AB confusion state) OR (AB cognitive impairment) OR (AB cognitive dysfunction) OR (AB psychomotor agitation) OR (AB psychotic disorders)  #3 #1 OR #2  #4 MH 'coronary artery bypass'  #5 (AB cardiopulmonary bypass) OR (AB aortocoronary bypass) OR (AB coronary surgery) OR (AB CABG) OR (AB open heart) OR (AB heart operation) OR (AB heart surgery) OR (AB heart surgical procedure) OR (AB cardiac surgery) OR (AB cardiac operation) OR (AB cardiac surgical procedures) OR (AB cardiovascular surgical procedure) OR (AB valv* surgery) OR (AB valve replacement) OR (AB congenital heart) OR (AB aortic dissection) OR (AB aneurysm)  #6 #4 OR #5  #7 #3 AND #6 | **September 5, 2021** | **610** |
| **Medline** | #1 (MeSH: "delirium") OR (TOPIC: "delirious") OR (TOPIC: "delusion") OR (TOPIC: "delusions") OR (TOPIC: "postoperative delirium") OR (TOPIC: "intensive care delirium") OR (TOPIC: "intensive care unit syndrome") OR (TOPIC: "intensive care delirium psychosis") OR (TOPIC: "ICU syndrome") OR (TOPIC: "ICU psychosis") OR (TOPIC: "ICU delirium") OR (TOPIC: "acute brain syndrome") OR (TOPIC: "acute confusional syndrome") OR (TOPIC: "acute confusion") OR (TOPIC: "confusion state") OR (TOPIC: "cognitive impairment") OR (TOPIC: "cognitive dysfunction") OR (TOPIC: "psychomotor agitation") OR (TOPIC: "psychotic disorders")  #2 (MESH: "coronary artery bypass") OR (TOPIC: "cardiopulmonary bypass") OR (TOPIC: "aortocoronary bypass") OR (TOPIC: "coronary surgery") OR (TOPIC: "CABG") OR (TOPIC: "open heart") OR (TOPIC: "heart operation") OR (TOPIC: "heart surgery") OR (TOPIC: "heart surgical procedure") OR (TOPIC: "cardiac surgery") OR (TOPIC: "cardiac operation") OR (TOPIC: "cardiac surgical procedures") OR (TOPIC: "cardiovascular surgical procedure") OR (TOPIC: "valv* surgery") OR (TOPIC: "valve replacement") OR (TOPIC: "congenital heart") OR (TOPIC: "aortic dissection") OR (TOPIC: "aneurysm")  3 #1 AND #2 | **September 15, 2021** | **5280** |
| **Wan-fang database**  **&**  **China National Knowledge Infrastructure** | #1主题:（心脏手术） OR 主题:（心脏外科） OR 主题:（冠状动脉搭桥术） OR 主题:（冠状动脉旁路移植术） OR 主题:（心脏瓣膜手术） OR 主题:（瓣膜置换术）OR 主题:（二尖瓣手术） OR 主题:（搭桥手术） OR 主题:（主动脉瓣手术） OR 主题:（瓣膜病） OR 主题:（体外循环手术）OR 主题:（主动脉夹层手术） OR 主题:（先天性心脏病） OR 主题:（动脉瘤）  #2主题:（谵妄） OR 主题:（术后谵妄） OR 主题:（认知功能障碍） OR主题:（精神障碍）OR 主题:（急性脑病综合征） OR 主题:（急性错乱状态） OR主题:（造影剂脑病）OR 主题:（ICU综合征） OR 主题:（ICU精神病）  3 #1 AND #2 | **September 20, 2021** | **2564** |

**Supplementary Table S2 Newcastle Ottawa Scale Ratings of the included studies**

| Author, Year | **Selection** | | | | **Comparability** | **Outcome** | | | **NOS** |
| --- | --- | --- | --- | --- | --- | --- | --- | --- | --- |
| Kati Järvelä 2017 | 🗸 | 🗸 | 🗸 | 🗸 |  | 🗸 | 🗸 | 🗸 | 7 |
| Andrea Kirfel  2016 | 🗸 | 🗸 | 🗸 |  | 🗸🗸 | 🗸 | 🗸 | 🗸 | 8 |
| Sandra Koster  2012 | 🗸 | 🗸 | 🗸 |  | 🗸🗸 | 🗸 | 🗸 |  | 7 |
| Katarzyna Kotfis  2018 | 🗸 | 🗸 | 🗸 | 🗸 | 🗸 | 🗸 | 🗸 |  | 7 |
| Kacper Lechowicz  2021 | 🗸 | 🗸 | 🗸 | 🗸 | 🗸 | 🗸 | 🗸 |  | 7 |
| Tania Luque  2021 | 🗸 | 🗸 | 🗸 | 🗸 | 🗸🗸 | 🗸 | 🗸 |  | 8 |
| Victor Mauri  2021 | 🗸 | 🗸 | 🗸 |  | 🗸🗸 | 🗸 | 🗸 |  | 7 |
| Dongliang Mu  2010 | 🗸 | 🗸 | 🗸 | 🗸 | 🗸 | 🗸 | 🗸 |  | 7 |
| Quyen Nguyen  2017 | 🗸 | 🗸 | 🗸 | 🗸 | 🗸 |  | 🗸 | 🗸 | 8 |
| Ieva Norkienė  2013 | 🗸 | 🗸 | 🗸 | 🗸 | 🗸 | 🗸 | 🗸 |  | 7 |
| Masato Ogawa  2017 | 🗸 | 🗸 | 🗸 |  | 🗸🗸 | 🗸 | 🗸 | 🗸 | 8 |
| Kamran Shadvar  2013 | 🗸 | 🗸 | 🗸 |  | 🗸 | 🗸 |  |  | 5 |
| Yukiharu Sugimura  2020 | 🗸 | 🗸 | 🗸 |  | 🗸 | 🗸 | 🗸 |  | 6 |
| Van der  2019 | 🗸 | 🗸 | 🗸 | 🗸 | 🗸🗸 | 🗸 | 🗸 |  | 8 |
| Charles H. Brown  2016 | 🗸 | 🗸 | 🗸 | 🗸 | 🗸 | 🗸 | 🗸 | 🗸 | 9 |
| Hersh S. Maniar  2015 | 🗸 | 🗸 | 🗸 | 🗸 | 🗸 | 🗸 | 🗸 |  | 7 |
| Sauër AC  2017 | 🗸 | 🗸 | 🗸 | 🗸 | 🗸 | 🗸 | 🗸 | 🗸 | 8 |
| Abla Habeeb-Allah  2019 | 🗸 | 🗸 | 🗸 | 🗸 | 🗸 | 🗸 | 🗸 |  | 7 |
| Stavros Theologou  2018 | 🗸 | 🗸 | 🗸 |  | 🗸 | 🗸 | 🗸 |  | 6 |
| Chetan P. Huded  2016 | 🗸 | 🗸 | 🗸 | 🗸 | 🗸 | 🗸 | 🗸 |  | 7 |
| Shining Cai  2020 | 🗸 | 🗸 | 🗸 | 🗸 | 🗸 | 🗸 | 🗸 | 🗸 | 8 |
| Sara J Beishuizen  2020 | 🗸 | 🗸 | 🗸 | 🗸 | 🗸 | 🗸 | 🗸 | 🗸 | 8 |
| Maciej Bagienski  2017 | 🗸 | 🗸 | 🗸 | 🗸 | 🗸🗸 | 🗸 | 🗸 |  | 8 |
| Masieh Abawi  2016 | 🗸 | 🗸 | 🗸 | 🗸 | 🗸 | 🗸 | 🗸 |  | 7 |
| Graciela Veliz-Reissmüller  2007 | 🗸 | 🗸 | 🗸 | 🗸 | 🗸 | 🗸 | 🗸 |  | 7 |
| Nina Smulter  2013 | 🗸 | 🗸 | 🗸 |  | 🗸 | 🗸 | 🗸 |  | 6 |
| Silvio Simeone  2018 | 🗸 | 🗸 | 🗸 | 🗸 | 🗸 | 🗸 | 🗸 |  | 7 |
| Gianfranco Sanson  2018 | 🗸 | 🗸 | 🗸 |  | 🗸🗸 | 🗸 | 🗸 | 🗸 | 8 |
| Franklin Santana Santos  2004 | 🗸 | 🗸 | 🗸 |  | 🗸 | 🗸 | 🗸 |  | 6 |
| Ieva Norkiene  2007 | 🗸 | 🗸 | 🗸 | 🗸 | 🗸 | 🗸 | 🗸 |  | 7 |
| Ashok K Kumar  2017 | 🗸 | 🗸 | 🗸 | 🗸 | 🗸 | 🗸 | 🗸 |  | 7 |
| Jakub Kazmierski  2010 | 🗸 | 🗸 | 🗸 | 🗸 | 🗸 | 🗸 | 🗸 |  | 7 |
| Yohei Kawatani  2015 |  | 🗸 | 🗸 | 🗸 | 🗸 | 🗸 | 🗸 |  | 6 |
| Robbert C. Bakker  2012 | 🗸 | 🗸 | 🗸 | 🗸 | 🗸 | 🗸 | 🗸 |  | 7 |
| Imran khan  2014 | 🗸 | 🗸 | 🗸 | 🗸 | 🗸 | 🗸 |  |  | 6 |
| Chaohong Chen  2018 | 🗸 | 🗸 | 🗸 |  | 🗸 | 🗸 | 🗸 |  | 6 |
| Liang Hong  2020 | 🗸 | 🗸 | 🗸 | 🗸 | 🗸 | 🗸 | 🗸 |  | 7 |
| Xianrong Song  2016 | 🗸 | 🗸 | 🗸 | 🗸 | 🗸 | 🗸 |  |  | 6 |
| Jian Wang  2020 | 🗸 | 🗸 | 🗸 | 🗸 | 🗸 | 🗸 | 🗸 |  | 7 |
| Qinying Wang  2019 | 🗸 | 🗸 | 🗸 | 🗸 | 🗸 | 🗸 | 🗸 |  | 7 |
| Qianyue Zhu  2020 | 🗸 | 🗸 | 🗸 |  | 🗸 | 🗸 | 🗸 |  | 6 |
| Lijing Su  2019 | 🗸 | 🗸 | 🗸 |  | 🗸 | 🗸 | 🗸 |  | 6 |

(A) Subgroup analysis in different study designs


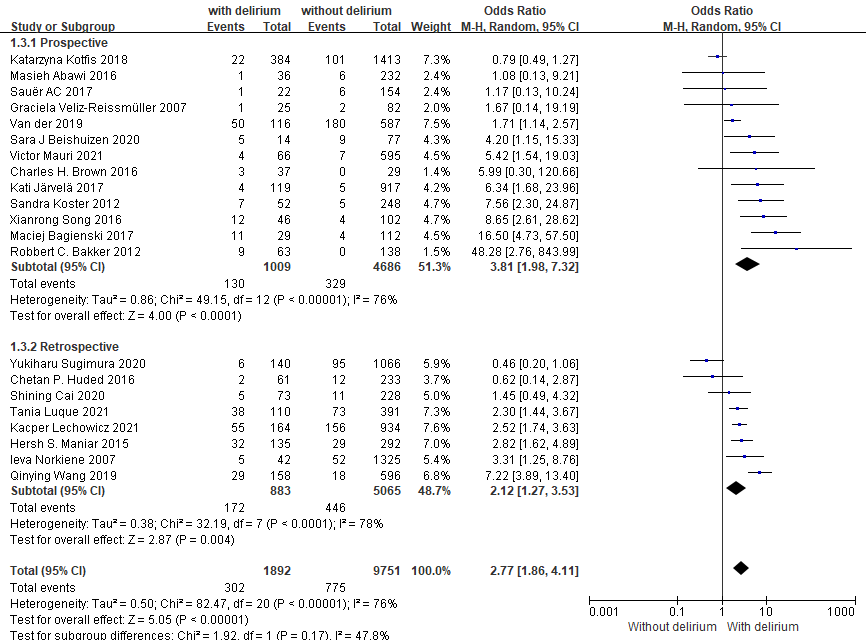


(B) Subgroup analysis in different sample sizes


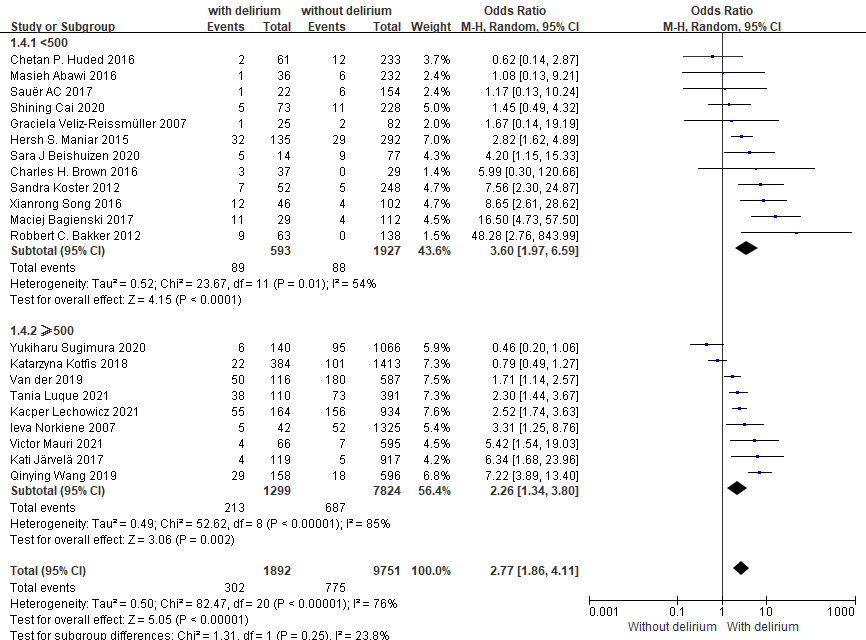


(C) Subgroup analysis in different male proportions


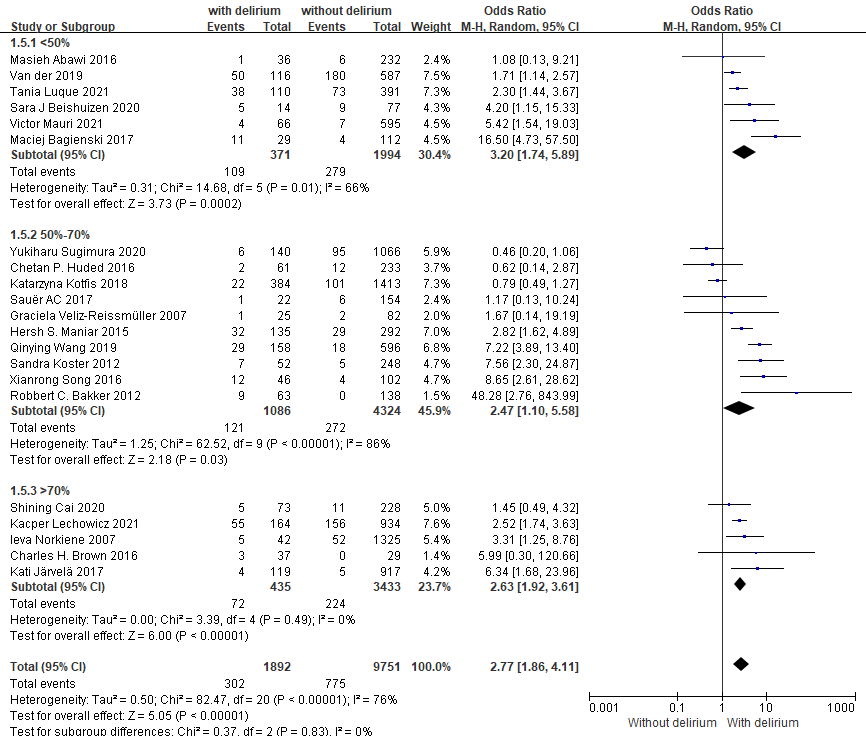


(D) Subgroup analysis in different surgery types


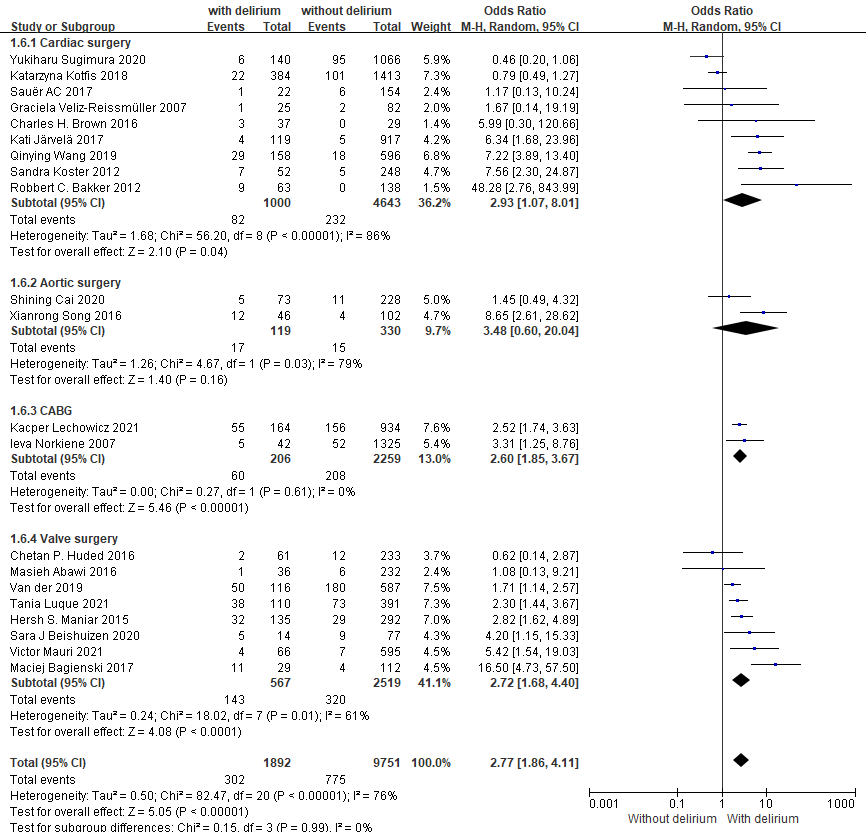


(E) Subgroup analysis in different countries


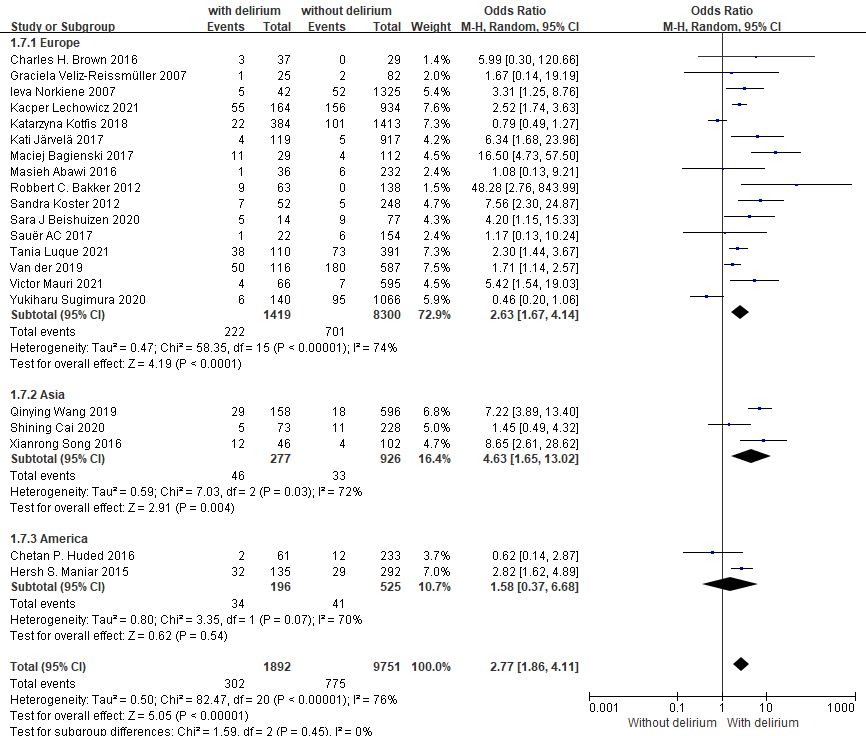


**Supplementary Figure S1. Subgroup analyses of the impact of postoperative delirium on mortality**

**Supplementary Table S3 Subgroup analysis of pooled OR for intubation time**

| **Categories** | **No. of studies** | **No. of patients** | **Pooled OR (95% CI)** | | **Heterogeneity** | |
| --- | --- | --- | --- | --- | --- | --- |
|  |  |  | **Random** | ***P*-value** | ***I²* (%)** | ***P*-value** |
| **Age** | **20** | **10469** | **0.83 (0.57, 1.09)** | **<.001** | **95** | **<.001** |
| <60 | 6 | 1745 | 1.27 (0.65, 1.89) | <.001 | 96 | <.001 |
| 60-70 | 9 | 6963 | 0.69 (0.43, 0.96) | <.001 | 88 | <.001 |
| >70 | 5 | 1761 | 0.53 (0.05, 1.01) | 0.030 | 93 | <.001 |
| **Study design** | **20** | **10469** | **0.83 (0.57, 1.09)** | **<.001** | **95** | **<.001** |
| Prospective | 15 | 4306 | 0.93 (0.57, 1.30) | <.001 | 96 | <.001 |
| Retrospective | 5 | 6163 | 0.56 (0.22, 0.90) | 0.001 | 93 | <.001 |
| **Operation time** | **20** | **10469** | **0.83 (0.57, 1.09)** | **<.001** | **95** | **<.001** |
| Elective | 8 | 2230 | 0.78 (0.28, 1.27) | 0.002 | 95 | <.001 |
| Emergency | 1 | 97 | 0.46 (0.13, 0.80) | 0.007 | N/A | N/A |
| Mixed | 5 | 3916 | 0.55 (0.17, 0.93) | 0.005 | 92 | <.001 |
| N/A | 6 | 4226 | 1.21 (0.64, 1.78) | <.001 | 97 | <.001 |
| **Male proportion** | **20** | **10469** | **0.83 (0.57, 1.09)** | **<.001** | **95** | **<.001** |
| <60% | 2 | 3287 | 0.98 (0.18, 1.77) | 0.020 | 91 | <.001 |
| 60%-70% | 7 | 2589 | 0.52 (0.13, 0.91) | 0.008 | 94 | <.001 |
| 70%-80% | 6 | 4006 | 0.73 (0.23, 1.23) | 0.004 | 96 | <.001 |
| >80% | 3 | 367 | 0.70 (0.44, 0.96) | <.001 | 30 | 0.240 |
| N/A | 2 | 220 | 2.36 (0.36, 4.36) | 0.020 | 96 | <.001 |
| **Sample size** | **20** | **10469** | **0.83 (0.57, 1.09)** | **<.001** | **95** | **<.001** |
| <500 | 13 | 1499 | 0.90 (0.49, 1.31) | <.001 | 93 | <.001 |
| ≥500 | 7 | 8970 | 0.72 (0.35, 1.10) | <.001 | 97 | <.001 |

OR, odds ratio; CI, confidence interval; N/A, not applicable.

**Supplementary Table S4 Subgroup analysis of pooled OR for ICU time**

| **Categories** | **No. of studies** | **No. of patients** | **Pooled OR (95% CI)** | | **Heterogeneity** | |
| --- | --- | --- | --- | --- | --- | --- |
|  |  |  | **Random** | ***P*-value** | ***I²* (%)** | ***P*-value** |
| **Study design** | **23** | **7398** | **0.87 (0.57, 1.17)** | **<.001** | **96** | **<.001** |
| Prospective | 17 | 4700 | 0.93 (0.54, 1.32) | <.001 | 97 | <.001 |
| Retrospective | 6 | 2698 | 0.70 (0.23, 1.18) | 0.004 | 95 | <.001 |
| **Sample size** | **23** | **7398** | **0.87 (0.57, 1.17)** | **<.001** | **96** | **<.001** |
| <500 | 17 | 2182 | 0.84 (0.57, 1.11) | <.001 | 89 | <.001 |
| ≥500 | 6 | 5216 | 0.94 (0.22, 1.66) | 0.010 | 99 | <.001 |
| **Operation time** | **23** | **7398** | **0.87 (0.57, 1.17)** | **<.001** | **96** | **<.001** |
| Elective | 8 | 1446 | 1.10 (0.38, 1.83) | 0.003 | 97 | <.001 |
| Mixed | 9 | 4826 | 0.74 (0.39, 1.08) | <.001 | 94 | <.001 |
| Emergency | 1 | 97 | 0.50 (0.16, 0.83) | 0.004 | N/A | N/A |
| N/A | 5 | 1029 | 0.81 (0.22, 1.41) | 0.007 | 95 | <.001 |
| **Region** | **23** | **7398** | **0.87 (0.57, 1.17)** | **0.001** | **96** | **<.001** |
| Asia | 11 | 2648 | 1.13 (0.46, 1.62) | <.001 | 97 | <.001 |
| North America | 1 | 29 | 0.85 (0.35, 1.36) | 0.001 | N/A | N/A |
| Europe | 11 | 4721 | 0.59 (0.31, 0.88) | <.001 | 92 | <.001 |

ICU, intensive care unit; OR, odds ratio; CI, confidence interval; N/A, not applicable.

**Supplementary Table S5 Subgroup analysis of pooled OR for hospital days**

| **Categories** | **No. of studies** | **No. of patients** | **Pooled OR (95% CI)** | | **Heterogeneity** | |
| --- | --- | --- | --- | --- | --- | --- |
|  |  |  | **Random** | ***P*-value** | ***I²* (%)** | ***P*-value** |
| **Region** | **19** | **6254** | **0.62 (0.48, 0.76)** | **<.001** | **81** | **<.001** |
| Asia | 5 | 711 | 0.71 (0.17, 1.25) | 0.010 | 90 | <.001 |
| Europe | 10 | 4974 | 0.53 (0.40, 0.66) | <.001 | 67 | 0.001 |
| North America | 4 | 569 | 0.85 (0.55, 1.14) | <.001 | 65 | 0.030 |
| **Studies design** | **19** | **6254** | **0.62 (0.48, 0.76)** | **<.001** | **81** | **<.001** |
| Prospective | 12 | 3244 | 0.67 (0.46, 0.88) | <.001 | 82 | <.001 |
| Retrospective | 7 | 3010 | 0.53 (0.33, 0.74) | <.001 | 78 | <.001 |
| **Sample size** | **19** | **6254** | **0.62 (0.48, 0.76)** | **<.001** | **81** | **<.001** |
| <100 | 3 | 175 | 0.41 (0.11, 0.71) | 0.008 | 0 | 0.540 |
| 100-200 | 6 | 731 | 0.64 (0.16, 1.12) | 0.009 | 90 | <.001 |
| 200-300 | 4 | 729 | 0.84 (0.58, 1.11) | <.001 | 66 | 0.030 |
| >300 | 6 | 4619 | 0.53 (0.40, 0.67) | <.001 | 71 | 0.004 |
| **Surgery type** | **19** | **6254** | **0.62 (0.48, 0.76)** | **<.001** | **81** | **<.001** |
| Cardiac surgery | 7 | 3208 | 0.50 (0.36, 0.64) | <.001 | 48 | 0.070 |
| Aortic surgery | 4 | 488 | 0.71 (0.03, 1.38) | 0.040 | 93 | <.001 |
| Valve surgery | 4 | 1288 | 0.75 (0.39, 1.11) | <.001 | 84 | <.001 |
| CABG | 4 | 1270 | 0.64 (0.43, 0.85) | <.001 | 50 | 0.110 |
| **Age** | **19** | **6254** | **0.62 (0.48, 0.76)** | **<.001** | **81** | **<.001** |
| <60 | 4 | 650 | 0.73 (0.07, 1.93) | 0.030 | 93 | <.001 |
| 60-70 | 6 | 2487 | 0.55 (0.39, 0.70) | <.001 | 45 | 0.110 |
| 70-80 | 5 | 1829 | 0.54 (0.34, 0.73) | <.001 | 59 | 0.040 |
| >80 | 4 | 1288 | 0.75 (0.39, 1.11) | <.001 | 84 | <.001 |
| **Male proportion** | **19** | **6254** | **0.62 (0.48, 0.76)** | **<.001** | **81** | **<.001** |
| <60% | 4 | 1288 | 0.75 (0.39, 1.11) | <.001 | 84 | <.001 |
| 60%-70% | 6 | 2962 | 0.71 (0.40, 1.03) | <.001 | 90 | <.001 |
| 70%-80% | 7 | 1720 | 0.46 (0.31, 0.62) | <.001 | 50 | 0.060 |
| >80% | 2 | 284 | 0.67 (0.34, 1.01) | <.001 | 0 | 0.770 |
| **CPB** | **19** | **6254** | **0.62 (0.48, 0.76)** | **<.001** | **81** | **<.001** |
| Yes | 15 | 5610 | 0.57 (0.42, 0.73) | <.001 | 81 | <.001 |
| Mixed | 1 | 223 | 0.72 (0.27, 1.16) | 0.002 | N/A | N/A |
| N/A | 3 | 421 | 0.83 (0.41, 1.24) | <.001 | 78 | 0.010 |

OR, odds ratio; CI, confidence interval; CPB, cardiopulmonary bypass; CABG, coronary artery bypass graft; N/A, not applicable.
